# The Impact of Mindfulness‐Based Counseling on the Mental Health of Women With a History of COVID‐19 During Pregnancy: A Quasi‐Experimental Study

**DOI:** 10.1002/brb3.70062

**Published:** 2024-09-30

**Authors:** Najmeh Shahriyari, Shabnam Omidvar, Farideh Mohsenzadeh‐Ledari, Alireza Azizi, Hemmat Gholinia

**Affiliations:** ^1^ Student Research Committee Babol University of Medical Sciences Babol Iran; ^2^ Social Determinants of Health Research Center, Health Research Institute Babol University of Medical Sciences Babol Iran; ^3^ Health Research Institute Babol Iran

**Keywords:** COVID‐19 | mental health | mindfulness | pregnancy

## Abstract

**Introduction:**

With the spread of COVID‐19, certain population groups, including pregnant women, were more susceptible than others. This disease can lead to postpartum complications, including mental disorders, in mothers. Few studies have investigated the impact of mindfulness‐based interventions on mental health, and the most effective counseling approach to promote mental health has not been identified.

**Objective:**

This study aimed to determine the impact of online mindfulness‐based counseling on improving mental health among women with a history of COVID‐19 during pregnancy in Iran.

**Methods:**

The present study was a quasi‐experimental design conducted on 100 women with a history of coronavirus infection during pregnancy referred to the Mother's Clinic of Yahya Nejad and Ayatollah Rouhani Educational‐Treatment Hospital, affiliated with Babol University of Medical Sciences, Iran, via convenience sampling. The women were randomly assigned to the intervention (mindfulness‐based counseling) and control groups. The intervention group received eight 45‐min weekly mindfulness‐based counseling sessions over 8 weeks. Data were collected via a demographic information questionnaire and the Goldberg General Health Questionnaire before and after the intervention, which were completed by both groups. Independent *t*‐tests and analysis of covariances (ANCOVAs) were used to compare the outcomes of the two groups.

**Results:**

After controlling for confounding variables, the mean mental health scores before and after counseling were 29.42 ± 4.49 and 19.80 ± 3.88, respectively, in the intervention group and 26.26 ± 2.29 and 25.92 ± 2.15, respectively, in the control group. The mean mental health score in the intervention group was significantly lower than that in the control group (*F* = 266.7, *p* < 0.001). The mean scores for somatic symptoms (*F* = 89.30, *p* < 0.001), depression symptoms (*F* = 142.71, *p* < 0.001), anxiety and insomnia symptoms (*F* = 120.56, *p* < 0.001), and social dysfunction scores (*F* = 127.77, *p* < 0.001) were significantly different between the two groups after counseling.

**Conclusion:**

The findings indicated that online mindfulness‐based counseling positively affects mental health and its domains during the postpartum period. However, further randomized clinical trials are needed before a definitive conclusion can be drawn.

**Trial Registration:**

We were not allowed to register according to the law of our country.

## Graphical abstract

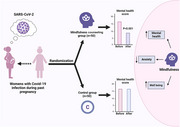

## 1 Introduction

Since the onset of the COVID‐19 outbreak, which was attributed to SARS‐CoV‐2, in December 2019 and continues through June 2022, the World Health Organization has reported more than 536 million confirmed cases and more than 6.3 million fatalities (Leroux 2022).

Throughout this time, efforts have been made to address mortality linked to this infection, and various infection prevention strategies and public policies have been implemented to reduce incidence rates and mitigate spread, achieving varying degrees of success (Chu et al. 2020). These initiatives resulted in significant disruptions to daily routines and social interactions. Nevertheless, despite these interventions, multiple waves and variants of the COVID‐19 pandemic have profoundly affected public health (Khoury et al. 2021; Ahorsu et al. 2022).

Studies have shown that COVID‐19 has caused an increase in mental disorders (depression, anxiety, and insomnia) in the general population (Huang and Zhao 2020; Moccia et al. 2020; Qiu et al. 2020; Voitsidis et al. 2020; Wang et al. 2020; Ahmed et al. 2022). Pandemic‐related stressors affect almost everyone, especially women (Almeida et al. 2020). Given that the occurrence of clinical manifestations, the intensity of symptoms, and the related obstetric complications appear to be heightened in comparison with the general population, pregnant women are regarded as a high‐risk group (Marchand et al. 2022; Allotey et al. 2020; Metz et al. 2022). Studies have also reported higher levels of anxiety and symptoms in postpartum women than in nonpregnant women of childbearing age (Fan et al. 2021; Farrell et al. 2020; Okagbue et al. 2019; Karimi, Khalili, and Nir 2020; Karimi et al. 2021).

A recent longitudinal study reported that at least 34.2% of obstetric patients with acute COVID‐19 infection presented post‐COVID‐19 symptoms. The most frequent findings were neurological and cutaneous manifestations (Muñoz‐Chápuli Gutiérrez et al. 2024).Other studies have also shown that COVID‐19 can be a risk factor for pregnancy complications (e.g., hypertensive disorders and preterm labor) (Allotey et al. 2020; Metz et al. 2022).

The clinical features associated with acute SARS‐CoV‐2 infection are widely recognized. Nevertheless, the various variants of the virus that have emerged during different waves of the pandemic may contribute to the range of symptoms observed (Tao et al. 2021). Additionally, the duration of recovery and the convalescence phase can vary significantly, and for those who survive COVID‐19, the illness may persist beyond the acute stage, leading to potential long‐term effects.

The conditions that arise following COVID‐19 pose a significant public health challenge in the aftermath of the pandemic, as the disease's pathology, potential diagnostic methods, and treatment targets remain inadequately understood. Recently, post‐COVID‐19 conditions have been characterized as new or ongoing common symptoms of COVID‐19 that manifest 3 months after the initial onset of the illness and persist for a minimum of 2 weeks, irrespective of the severity experienced during the acute phase. The identified risk factors include female gender, obesity, prior psychiatric conditions, and the total number of symptoms present at the onset of the disease (Yong 2021; Michelen et al. 2021; Akbarialiabad et al. 2021). Another study from Iran revealed that during the COVID‐19 pandemic, pregnant women faced significant stress and anxiety stemming from various factors, including quarantine measures, limited access to healthcare services, separation from friends and family, and pervasive feelings of loneliness. Additionally, the reliance on avoidance coping strategies to manage stress adversely affects mental well‐being (Firouzbakht et al. 2022). However, assessing post‐COVID‐19 conditions is limited. One of the methods available to improve mental health during the post‐COVID‐19 pandemic is mindfulness‐based interventions (MBIs), including educational mind‐body programs that aim to train the mind through medication to achieve nonjudgmental awareness focused on the present moment (Oskoui et al. 2023; Sacristan‐Martin et al. 2019). As this approach benefits people with depression and other mental health disorders (Goldberg et al. 2018), learning and practicing mindfulness skills during pregnancy can improve the mother's depression symptoms and even the baby's birth weight (Oskoui et al. 2023; Nyklíček et al. 2018).

Based on a review of studies conducted in Iran, no mindfulness‐based counseling interventions have been conducted on the mental health of women with a history of COVID‐19 during pregnancy. Given the importance of this issue, the conditions of society, the great importance of social distancing in preventing coronavirus infection, cost reduction, more accessible access, better time management, and increased peace of mind during learning, virtual intervention (online) was preferable to in‐person intervention. Therefore, this study was conducted to determine the impact of mindfulness‐based group counseling on the mental health of women with a history of COVID‐19 during pregnancy.

### 1.1 Objective

To evaluate the impact of online mindfulness‐based counseling on improving mental health among women with a history of COVID‐19 during pregnancy in Iran.

## 2 Materials and Methods

The participants were randomly assigned to either the intervention (mindfulness‐based counseling) or control group. The intervention group received eight 45‐min weekly mindfulness‐based counseling sessions over 8 weeks led by the researcher via a social network. Data were collected via a demographic information questionnaire and the Goldberg General Health Questionnaire (GHQ‐28) before and after the intervention, which were completed by both groups. Independent *t*‐tests and analysis of covariances (ANCOVAs) were used to compare the outcomes between the two groups.

### 2.1 Study Design and Participants

We designed a hospital‐based intervention study including 100 pregnant women enrolled in a COVID‐19 and pregnancy program at a tertiary referral center in Iran (Babol). All women included were referred to the program because of a confirmed positive COVID‐19 test during pregnancy or at admission for labor, after discharge from the emergency room, after hospitalization, or after the confirmation of COVID‐19 infection in obstetric or primary care consultations.

According to WHO recommendations, confirmed SARS‐CoV‐2 infection was defined as a positive result from a real‐time reverse transcriptase‐polymerase chain reaction (RT‐PCR) assay of a nasopharyngeal swab. We included pregnant women with positive SARS‐CoV‐2 RT‐PCR results from November 2021 to February 2022. All patients provided verbal consent to participate in the follow‐up study, and they did not present significant language or communication barriers.

The inclusion criteria included those who were willing to participate in the study, were reading and writing literacy, had access to social networks via a cell phone, without mental disabilities or deafness, had no history of mental health problems, no drug addiction, no use of antianxiety and stress medications, no alcohol use, no participation in yoga and mindfulness classes in the last 6 months, who gave birth in the aforementioned hospitals, and had a score > 23 and < 60 on the GHQ‐28 questionnaire. The exclusion criteria included recent bereavement, failure to consent to continue the study, and failure to complete two or more sessions from eight mindfulness‐based counseling sessions. This study was a master's thesis, which investigated the effect of mindfulness‐based counseling on mental health in women with a history of coronavirus disease (COVID‐19) during pregnancy.

### 2.2 Sample Size

The sample size was determined on the basis of the mean score of mental health, and according to the study of Pan et al. (2019), considering that *σ*1 =  6.25 (mean score of mental health), *σ*2 =  5.76 (assuming a 25% increase due to the intervention), *d* =  3.5, *α* =  0.05, *β* = 0.2, and power =  80%, the sample size was estimated to be 42 subjects in each group. Finally, the sample size was considered 50 in each group on the basis of the variables of mental health and domains and regarding 10% attrition.

### 2.3 Sampling and Random Assignment

This study included women referred to the program because of a confirmed positive COVID‐19 test during pregnancy or at admission for labor, after discharge from the emergency room, after hospitalization, or after the confirmation of COVID‐19 infection in obstetric or primary care consultations to the Mothers’ Clinic of Yahya Nejad and Ayatollah Rouhani Educational‐Treatment Hospital, affiliated with Babol University of Medical Sciences, Iran. The researcher (the first author, who was a senior student in midwifery counseling and had a certificate in a mindfulness‐based counseling course) attended the Deputy of Treatment of the aforementioned university and reviewed the inclusion criteria.

**FIGURE 1:**
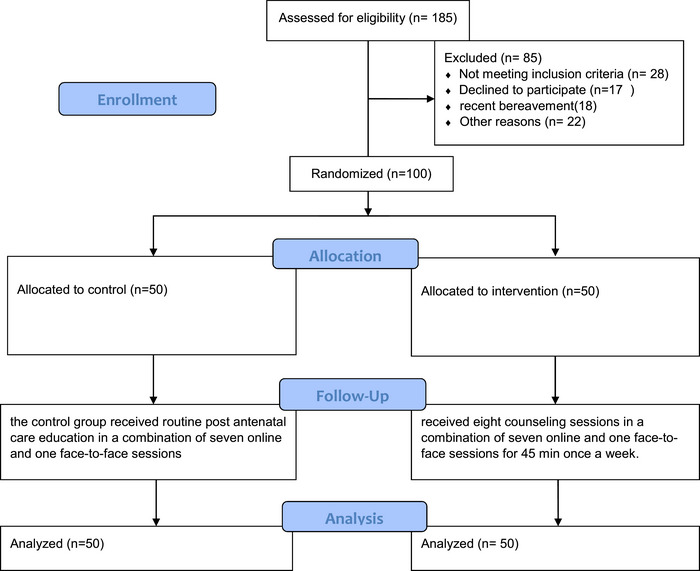
CONSORT flow diagram of patients included in the study.

The eligible women were requested to attend a face‐to‐face session, and the objectives and methods of the study were fully explained. The written informed consent form was subsequently obtained from the women willing to participate in the research, and the sociodemographic and obstetric characteristics questionnaire was completed. Initially, 185 people were selected, but 85 withdrew from the study for personal reasons, leaving 100 participants who accepted the study conditions (Figure 1). In this study, the participants were selected via convenience sampling. They were assigned to the intervention (mindfulness‐based counseling) and control groups at a ratio of 1:1 by blocked randomization via random allocation software (RAS) with a block size of 4. Blocking was performed by a noninvolved person during sampling and data analysis. Intervention sequences were written on 100 cards via the randomization list to ensure blinding, with the corresponding coding known only to the study supervisor. When an eligible patient was identified, the study supervisor informed her of the nature of the intervention. However, blinding was not possible due to the nature of the intervention modalities in the two groups, so the study used an open‐label design.

### 2.4 Mindfulness‐Based Counseling Intervention

According to Nancy Bardacke and the MBCP and MBSR programs, the mindfulness‐based counseling program, consisting of eight 45‐min educational sessions, was developed and approved by experts in psychology and midwifery.

The intervention group (the mindfulness receiving group) received eight counseling sessions in a combination of seven online sessions and one face‐to‐face session for 45 min once a week. The researcher checked the homework requested from the mothers, and feedback was given to the mothers. The control group did not receive any counseling training during the study (Table 1).

**TABLE 1:** The content of the consultations.

Topic	Sessions
Session 1 (automatic performance and overcoming obstacles)	Brief description of five therapy sessions and the necessity and logic behind mindfulness Therapy, performing meditation by eating a raisin, scanning the body for 30 min and discussing the emotions arising from this scan, performing a seated meditation, presenting the task of eating a raisin and body scanning and practicing mindfulness in daily activities as well as examining the obstacles to performing this exercise
Session 2 (mindful breathing and staying in the moment)	Gentle mindful movements to soothe somatic symptoms, 2 min of nonjudgmental seeing and hearing practice, seated meditation, and breathing with attention to bodily sensations
Session 3 (acceptance and allowing)	Presenting and performing mindful body movements and seated meditation, presenting a mood exercise, thinking with separate perspectives with the understanding that most thoughts are not real, a 3‐min breathing space exercise, having meditation exercises, and choosing a combination of meditations based on personal preference for home practice, as well as performing a 3‐min breathing space exercise in an unpleasant event and exercising mindfulness in a new daily activity
Session 4	Reviewing the four‐dimensional meditation, finding the best way for self‐care, setting a plan with sufficient positive approaches, having a 3‐min breathing space, and discussing ways to deal with meditation obstacles
Sessions 5–8	Reviewing previous session exercises and requesting participants to apply all mindfulness techniques to reduce momentary stress and perform four‐dimensional meditations

### 2.5 Data Collection Tools

The data were collected via a demographic and obstetric characteristics questionnaire and a mental health questionnaire. The demographic and obstetric characteristics questionnaire was completed before the intervention in both groups, and the mental health questionnaire with the GHQ‐28 questionnaire was completed by both groups before and after the intervention.

### 2.6 Demographic and Obstetric Characteristics Questionnaire

The questionnaire included the variables of age, education, occupation, family income, and so on.

Content and face validity were used to determine the validity of the demographic and obstetrics characteristics questionnaire. The questionnaire was given to the faculty members. On the basis of the feedback received, corrections were made to the tools after their opinions were collected.

### 2.7 The Goldberg GHQ‐28

This 28‐item questionnaire was developed by Hjelle et al. (2019) to measure mental health and four main domains of mental disorders, including somatic symptoms, anxiety and insomnia, social dysfunction, and depression. A participant receives five scores: four related to the subscales, and one is the total score. The score for each individual on each subscale ranges from 0 to 21, and the total score ranges from 0 to 84. Many studies have reviewed and confirmed the reliability and validity of this questionnaire (Pan et al. 2019; Nazifi et al. 2013; Hjelle et al. 2019; Sterling 2011; Farahbakhsh and Dehghani 2017; Dai et al. 2022; Si et al. 2021; Cejudo et al. 2019).

### 2.8 Statistical Analysis

Statistical analysis was performed via SPSS version 26. The descriptive statistics included the means and standard deviations for quantitative data and the frequencies and percentages for qualitative data. Data normality was tested via the Kolmogorov–Smirnov test. To examine the difference between the variables at the start of the study (baseline variables), the *t*‐test and the chi‐square test were used; in the case of non‐normality, their nonparametric equivalent, the Mann–Whitney *U*–test, and Fisher's exact test were employed. ANCOVA was used to examine and analyze the effects of the intervention variables in the two study groups. The results were calculated and reported with a 95% confidence interval and a significance level of 0.05.

## 3 Results

Sampling started in November 2021 and continued until January 2022. Initially, 140 people were selected, but 40 withdrew from the study for personal reasons, leaving 100 participants who accepted the study conditions. Overall, 100 participants were recruited and randomly assigned to the intervention (50 participants) and control (50 participants) groups via a random list, and they received eight counseling sessions. The women were assessed, and the data were analyzed after the intervention (Figure 1). The demographic characteristics of the two groups are compared in Table 2. The results revealed no significant differences between the participants in the two groups in terms of demographic characteristics.

**TABLE 2:** Demographic and obstetric characteristics of the participants.

Maternal characteristics	Intervention group (*N* = 50) Number (%)	Control group (*N* = 50) Number (%)	*p* value
Mother's age (year)	30.48 ± 4.36	29.98 ± 3.49	0.638
The interval between sampling and disease diagnosis (months)	8.05 ± 3.24	8.05 ± 2.09	0.943
Time of disease			
First trimester	12 (24)	9 (18)	0.649
Second trimester	21 (42)	23 (46)	
Third trimester	17 (34)	18 (36)	
Education			
< Diploma	19 (38)	10 (20)	0.250
≥ Diploma	31 (62)	40 (80)	
Economic status			
Low	9 (18)	8 (16)	0.920
Middle/Good	41 (82)	42 (84)	
Gravida			
≤ 2	42 (84)	45 (90)	0.117
> 2	8 (16)	5 (10)	
Parity			
1	22 (44)	24 (48)	0.135
> 1	28 (56)	26 (52)	
Planned pregnancy			
No	11 (22)	8 (16)	0.444
Yes	39 (78)	42 (84)	
Job			
Employee	6 (12)	8 (16)	0.17
Freelancer	5 (10)	11 (22)	
Unemployed	39 (78)	31 (62)	
Hometown			
City	27 (54)	34 (68)	0.151
Village	23 (46)	16 (32)	

In the study of the mental health variable, in the intervention group, the mean of this variable decreased by 9.6 compared with that before the intervention, which showed that this value was significant (*p* < 0.001), whereas this difference in the control group was 0.34, which was not significantly different (*p* = 0.271). Before the intervention, there was a significant difference in the average mental health variables between the two intervention groups and the control group (*p* < 0.001) (Table 3).

**TABLE 3:** Comparison of the means of the mental health variable and its subscales in the two study groups before and after the intervention.

	Groups	*N*	Before (mean ± SD)	After (mean ± SD)	^a^MD	*p* value^b^ (within‐subjects)
Mental health	Intervention group	50	29.42 ± 4.99	19.80 ± 3.88	9.62	< 0.001
	Control group	50	26.26 ± 2.29	25.92 ± 2.15	0.34	0.271
	*p* value^c^ (between‐subjects)		< 0.001	—	—	—
Somatic symptoms	Intervention group	50	6.90 ± 1.87	4.96 ± 1.54	1.94	< 0.001
	Control group	50	6.24 ± 0.72	6.22 ± 0.98	0.02	0.864
	*p* value^c^ (between‐subjects)		0.023	—	—	—
Anxiety and insomnia	Intervention group	50	8.14 ± 1.53	5.28 ± 1.05	2.86	< 0.001
	Control group	50	7.10 ± 0.95	6.70 ± 1.07	0.40	0.002
	*p* value^c^ (between‐subjects)		< 0.001		—	—
Social dysfunction	Intervention group	50	6.92 ± 1.68	4.72 ± 1.39	2.20	< 0.001
	Control group	50	6.44 ± 0.91	6.56 ± 0.84	0.12	0.293
	*p* value^c^ (between‐subjects)		0.079	—	—	—
Depression	Intervention group	50	7.42 ± 1.61	4.76 ± 1.19	2.66	< 0.001
	Control group	50	6.30 ± 1.17	6.34 ± 1.06	0.04	0.785
	*p* value^c^ (between‐subjects)		< 0.001	—	—	—

^a^ Mean difference.

^b^ Paired *t‐*test.

^c^ Independent *t‐*test.

Additionally, ANCOVA was used for comparisons between groups after the intervention. One way to control the effects of scores before the intervention in two groups is to examine the effects of the intervention separately from the potential effect of the scores before the intervention and to answer the question of whether the average of the adjusted group is significantly different from each other. In this type of analysis, more reliable results are provided, and by increasing the validity of the results, the generalizability of the study also increases. Before conducting the covariance analysis test, the assumptions of the test, including the equality of the dependent variable variances in the groups, were confirmed through the Lon test, and the homogeneity of the regression slope was also confirmed through the regression line slope homogeneity test.

According to the results listed in Table 3, the mean mental health variable in the intervention group was significantly lower than that in the control group (*F* = 266.7, *p* < 0.001). The effectiveness of the intervention was calculated (*η* ^2^ = 0.733). In other words, 73.3% of the explained variance in the adjusted mean of the quality variable is caused by the intervention. The statistical power in this case was reported as 0.99 (Table 4).

**TABLE 4:** The results of the analysis of covariance (ANCOVA) comparing the scores before and after the intervention of the mental health variable and its subscales in the two study groups before and after the intervention.

	Source	Type III sum of squares	df	Mean square	*F*	*p* value	Partial *η* ^2^	Observed power
Mental health	Mental health (before intervention)	478.81	1	478.81	94.6	< 0.001	0.494	1.00
	Groups	1338	1	1338	266.7	< 0.001	0.733	1.00
Somatic symptoms	Somatic symptoms (before intervention)	87.94	1	87.94	114.40	< 0.001	0.541	1.00
	Groups	68.64	1	68.64	89.30	< 0.001	0.479	1.00
Anxiety and insomnia	Anxiety and insomnia (before intervention)	44.02	1	44.02	64.16	< 0.001	0.398	1.00
	Groups	82.72	1	82.72	120.56	< 0.001	0.554	1.00
Social dysfunction	Social dysfunction (before intervention)	48.13	1	48.13	58.16	< 0.001	0.375	1.00
	Groups	106.73	1	106.73	127.77	< 0.001	0.568	1.00
Depression	Depression (before intervention)	54.99	1	54.99	76.93	< 0.001	0.442	1.00
	Groups	102.02	1	102.02	142.71	< 0.001	0.596	1.00

## 4 Discussion

These findings are consistent with those of Farahbakhsh and Dehghani (2017), who reported the effectiveness of mindfulness therapy on sleep quality and mental health in women with insomnia, indicating improved mental health (Farahbakhsh and Dehghani 2017). A study by Dai et al. (2022), which investigated the effectiveness of virtual mindfulness interventions on the mental health of nursing students during the coronavirus pandemic in China, and a study by Si et al. (2021), investigated the effects of virtual mindfulness intervention training on the mental health and quality of life of patients with COVID‐19, reported a reduced mean mental health score, indicating improvements in mental health in the experimental group (Dai et al. 2022; Si et al. 2021). In addition, the study results were also consistent with those of Cejudo et al. (2019). On the other hand, the results related to the improvement of mental health domains (somatic symptoms, anxiety and insomnia, depression, and social dysfunction) were consistent with the results of Shoaa Kazemi (2017), who investigated the efficacy of mindfulness‐based cognitive processes in improving the mental health of betrayed women. However, the most considerable change in their study was related to the depression domain, whereas the most significant change in our findings was related to the anxiety and insomnia domains (Shoaa Kazemi 2017; Innab et al. 2023). The discrepancies in the results could be due to differences in the target group, that is, their study investigated betrayed women. Given the consistency of the results of this study with those of similar studies, it can be claimed that counseling can reduce stress and anxiety and alleviate depression by building trust, empathy, and good communication between clients and counselors in a peaceful environment. The foundation of mindfulness‐based therapy is the presence of the mind at every moment, preventing rumination, taking control of daily events, recognizing automatic thought patterns focused on breathing, increasing concentration and integration, recognizing false cognitions, and accepting thoughts and dealing with them. Mindfulness refers to paying attention in a certain way, focusing on a goal, in the present moment, and without judgment. It means creating awareness in individuals about perceptions, cognitions, emotions, or feelings without judging or evaluating them as real or false, healthy or unhealthy, and important or unimportant. This method can help reduce various problematic conditions, such as the recurrence of stress, anxiety, and depression, and maintain an individual's mental health. Mindfulness‐based counseling helps individuals gain awareness of repetitive cognitive patterns employed to cope with thoughts and emotions related to COVID‐19 that have led to stress and emotional distress. By attending mindfulness‐based counseling sessions, women learn to change and control negative attitudes and engage less in distressing and self‐destructive thoughts, resulting in reduced anxiety and depression and improved mental health. Another process that helps improve women's mental health is unjudgmental attention to events. Their negative attitudes decrease when they try to accept all thoughts and related events without judgment or avoidance.

### 4.1 Strengths and Limitations

Some of the strengths of the present study included conducting an interventional study with random allocation, providing mindfulness‐based counseling by a researcher who had a certificate of mindfulness skills in the presence of a clinical psychologist, and using valid questionnaires whose psychometric properties have already been evaluated in Iran. Given that the present study was conducted during the COVID‐19 pandemic, the researcher had no meetings with participants, except for the first face‐to‐face session, due to the design and nature of the study and the simultaneous implementation of the project with the outbreak and spread of the coronavirus disease. As a result, less participation, collaboration, responsiveness, and research engagement were observed during project implementation, resulting in a relatively higher percentage of sample attrition. Another limitation was the inability of the researcher to fully monitor the proper implementation of the guidelines.

## 5 Conclusion

This study is the first to investigate the effects of MBIs on the mental health of women. The results indicated that mindfulness‐based counseling has a positive effect on mental health. Accordingly, women can better manage mental disorders postpartum via mindfulness. The results of the present study can help researchers design and present different effective care models, including psychological care and consultations. Furthermore, it is recommended that maternal health policymakers and planners make decisions on providing trained midwives with appropriate counseling skills to provide the necessary care for women during the postpartum period along with mindfulness‐based education.

## Author Contributions

**Najmeh Shahriyari**: conceptualization, data curation, writing–original draft, investigation. **Shabnam Omidvar**: conceptualization, writing–review and editing, supervision, project administration. **Farideh Mohsenzadeh‐Ledari**: conceptualization, supervision, writing–original draft, writing–review and editing, investigation, methodology, project administration, software, data curation, formal analysis. **Alireza Azizi**: writing–review and editing. **Hemmat Gholinia**: formal analysis, writing–review and editing.

## Ethics Statement

This research was approved by the Ethics Committee of the Deputy of Research and Technology at the Babol University of Medical Sciences, Babol, Iran (code number: IR. MU BABOL.REC.1400.252) on November 21, 2021.

## Consent

All participants were assured of the matter of confidentiality. Additionally, informed written consent was obtained from all participants. All methods were performed in accordance with the Declaration of Helsinki.

## Conflicts of Interest

The authors declare no conflicts of interest.

### Peer Review

The peer review history for this article is available at https://publons.com/publon/10.1002/brb3.70062.

## Data Availability

The datasets used and/or analyzed during the current study are available from the corresponding author upon reasonable request.
